# Clinical significance of high-dose cytarabine added to cyclophosphamide/total-body irradiation in bone marrow or peripheral blood stem cell transplantation for myeloid malignancy

**DOI:** 10.1186/s13045-015-0201-x

**Published:** 2015-09-04

**Authors:** Yasuyuki Arai, Kazunari Aoki, June Takeda, Tadakazu Kondo, Tetsuya Eto, Shuichi Ota, Hisako Hashimoto, Takahiro Fukuda, Yukiyasu Ozawa, Yoshinobu Kanda, Chiaki Kato, Mineo Kurokawa, Koji Iwato, Makoto Onizuka, Tatsuo Ichinohe, Yoshiko Atsuta, Akiyoshi Takami, on behalf of the AML and MDS Working Group of the Japan Society for Hematopoietic Cell Transplantation

**Affiliations:** Department of Hematology and Oncology, Graduate School of Medicine, Kyoto University, 54, Shogoin Kawahara-cho, Sakyo-ku, Kyoto 606-8507 Japan; Department of Hematology, Hamanomachi Hospital, Fukuoka, Japan; Department of Hematology, Sapporo Hokuyu Hospital, Sapporo, Japan; Department of Hematology/Division of Stem Cell Transplantation, Kobe General Hospital/Institute of Biomedical Research and Innovation, Kobe, Japan; Department of Hematopoietic Stem Cell Transplantation, National Cancer Center Hospital, Tokyo, Japan; Department of Hematology, Japanese Red Cross Nagoya First Hospital, Nagoya, Japan; Division of Hematology, Saitama Medical Center, Jichi Medical University, Saitama, Japan; Department of Hematology, Meitetsu Hospital, Nagoya, Japan; Department of Cell Therapy and Transplantation Medicine, the University of Tokyo Hospital, Tokyo, Japan; Department of Hematology, Hiroshima Red Cross Hospital & Atomic-bomb Survivors Hospital, Hiroshima, Japan; Department of Hematology/Oncology, Tokai University School of Medicine, Isehara, Japan; Department of Hematology and Oncology, Hiroshima University Hospital, Hiroshima, Japan; Japanese Data Center for Hematopoietic Cell Transplantation, Nagoya, Japan; Department of Healthcare Administration, Nagoya University Graduate School of Medicine, Nagoya, Japan; Department of Internal Medicine Division of Hematology, Aichi Medical University, Nagakute, Japan

## Abstract

**Background:**

Addition of high-dose cytarabine (HDCA) to the conventional cyclophosphamide/total-body irradiation (CY/TBI) regimen significantly improved prognosis after cord blood transplantation (CBT) for adult acute myelogenous leukemia (AML) and myelodysplastic syndrome (MDS). The efficacy of HDCA in bone marrow or peripheral blood stem cell transplantation (BMT/PBSCT), however, has not yet been elucidated.

**Findings:**

We conducted a cohort study to compare the prognosis of HDCA/CY/TBI (*N* = 435) and CY/TBI (*N* = 1667) in BMT/PBSCT for AML/MDS using a Japanese transplant registry database. The median age was 38 years, and 86.0 % of the patients had AML. Unrelated donors comprised 54.6 %, and 63.9 % of donors were human leukocyte antigen (HLA)-matched. Overall survival (OS) was not improved in the HDCA/CY/TBI group (adjusted hazard ratio (HR), 1.14; *p* = 0.13). Neutrophil engraftment was inferior (HR, 0.80; *p* < 0.01), and the incidence of hemorrhagic cystitis and thrombotic microangiopathy increased in HDCA/CY/TBI (HR, 1.47 and 1.60; *p* = 0.06 and 0.04, respectively), leading to significantly higher non-relapse mortality (NRM; HR, 1.48; *p* < 0.01). Post-transplant relapse and tumor-related mortality were not suppressed by the addition of HDCA.

**Conclusions:**

This study indicated the inefficacy of HDCA/CY/TBI in BMT/PBSCT for AML/MDS. Our results should be validated in large-scale prospective studies.

**Electronic supplementary material:**

The online version of this article (doi:10.1186/s13045-015-0201-x) contains supplementary material, which is available to authorized users.

## Introduction

Cyclophosphamide/total-body irradiation (CY/TBI) is a widely known conventional myeloablative regimen in allogeneic hematopoietic cell transplantation (HCT) for adult acute myelogenous leukemia (AML) and myelodysplastic syndrome (MDS) [1–3], while regimens with stronger anti-leukemic effects have been sought to reduce post-transplant relapse [4]. Among them, the addition of high-dose cytarabine (HDCA) to CY/TBI may be promising; in our recent large-scale study, HDCA/CY/TBI significantly improved overall survival (OS) compared to CY/TBI by suppressing relapse without increasing severe adverse events or non-relapse mortality (NRM) in cord blood transplantation (CBT) for AML/MDS [5]. However, previous studies in a small cohort with mixed hematopoietic malignancies showed that HDCA/CY/TBI increased NRM after bone marrow transplantation (BMT) [6, 7]. These results require validation using disease-specified and newer cohorts, in order to reflect the characteristics of each malignancy and the recent progress in supportive therapies, such as antibiotics. Therefore, we performed a cohort study to compare prognosis following HDCA/CY/TBI and CY/TBI in AML/MDS patients who underwent BMT or peripheral blood stem cell transplantation (PBSCT), using the Japanese transplant registry database.

## Patients and methods

Data for adult patients (age ≥16 years) with AML and MDS who underwent allogeneic BMT or PBSCT from related (Rel) or unrelated (UR) donors as first HCT after CY/TBI (CY, total 120 mg/kg; TBI, 10–12 Gy) or HDCA/CY/TBI (CA, 2–3 g/m^2^ twice a day for 2–3 days) [5] between January 1, 2000 and December 31, 2012, were obtained from the Japanese Transplant Registry Unified Management Program (TRUMP) [8]. UR-PBSCT and haploidentical HCT were not included because of the small number of patients. Donor-derived serum and/or erythrocytes were depleted from grafts in case of mismatched ABO blood type, and grafts were transplanted without T cell depletion. Our protocol complied with the Declaration of Helsinki, and it was approved by the TRUMP Data Management Committee and the Ethics Committee of Kyoto University. Each patient provided written informed consent.

From the registry database, we extracted data on basic pre-transplant characteristics and post-transplant clinical courses. Disease risk was defined as previously reported [9]. Disparity in human leukocyte antigen (HLA)-A, B, and DR antigens was determined at the serologic level in Rel-BMT and Rel-PBSCT. In UR-BMT, 8 antigens including HLA-C were determined at the allele level; a 6/6 (Rel) or 8/8 (UR) match was considered HLA-matched [10]. Statistical analyses were performed as described previously [5].

## Results

We evaluated 2102 patients who underwent HCT with CY/TBI (*N* = 1667) or HDCA/CY/TBI (*N* = 435), with a median follow-up of 1134 days (range, 40–4947 days). Patients with AML and high-risk disease were conditioned more frequently with HDCA/CY/TBI (Table 1). The dose of TBI was not different between CY/TBI and HDCA/CY/TBI (10 Gy, 4.5 vs 4.3 %; 12 Gy, 95.1 vs 94.3 %, respectively); 12 Gy was divided into 4 (26.1 vs 34.1 %) or 6 fractions (70.2 vs 60.9 %). Graft-versus-host disease (GVHD) prophylaxis composed of cyclosporine (49.1 %) or tacrolimus (50.9 %), most of which (more than 96 %) were combined with methotrexate. No difference was observed between the two groups.

**Table 1:** Patient characteristics

Variables		Total		CY/TBI		HDCA/CY/TBI		
		*N* = 2102	%	*N* = 1667	%	*N* = 435	%	*p*
Sex	Male	1205	57.3	959	57.5	246	56.5	
	Female	897	42.7	708	42.5	189	43.5	0.71
Age	Median (years)	38		38		38		0.10
	(Range)	(16–64)		(16–62)		(16–64)		
	≤49	1762	83.8	1391	83.4	371	85.3	
	≥50	340	16.2	276	16.6	64	14.7	0.35
PS	0–1	1963	93.3	1577	94.6	386	88.7	
	≥2	104	5.0	62	3.7	42	9.7	
	Unknown	35	1.7	28	1.7	7	1.6	<0.01^*^
HCT-CI	≤2	1287	61.2	1024	61.4	263	60.5	
	≥3	111	5.3	90	5.4	21	4.8	
	Unknown	704	33.5	553	33.2	151	34.7	0.78
CMV sero-status	Negative	392	18.7	312	18.7	80	18.4	
	Positive	1524	72.4	1221	73.3	303	69.6	
	Unknown	186	8.9	134	8.0	52	12.0	0.04^*^
Diagnosis	AML	1808	86.0	1397	83.8	411	94.5	
	MDS	294	14.0	270	16.2	24	5.5	<0.01^*^
Disease risk	Standard	1276	60.7	1074	64.4	202	46.4	
	High	826	39.3	593	35.6	233	53.6	<0.01^*^
(In AML)	Standard	1191	65.9	993	71.1	198	48.2	
	High	617	34.1	404	28.9	213	51.8	<0.01^*^
(In MDS)	Standard	85	28.9	81	30.0	4	16.7	
	High	209	71.1	189	70.0	20	83.3	0.17
Days from diagnosis to HCT	Median	239		240		237		0.03^*^
	≤240	1056	50.2	834	50.0	222	51.0	
	≥241	1046	49.8	833	50.0	213	49.0	0.71
Donor source	Rel-BM	455	21.7	351	21.1	104	23.9	
	Rel-PB	499	23.7	386	23.2	113	26.0	
	UR-BM	1148	54.6	930	55.7	218	50.1	0.11
Graft cell dose	BM (NCC, median)	2.69 × 10^8^/kg		2.66 × 10^8^/kg		2.77 × 10^8^/kg		0.27
	PB (CD34^+^ cell count, median)	3.99× 10^6^/kg		4.00× 10^6^/kg		3.67× 10^6^/kg		0.52
HLA mismatch	Matched	1343	63.9	1057	63.4	286	65.7	
	Mismatched	759	36.1	610	36.6	149	34.3	0.37
Sex mismatch	Matched	1145	54.4	919	55.1	226	51.9	
	M to F	508	24.2	398	23.9	110	25.3	
	F to M	445	21.2	346	20.8	99	22.8	
	Unknown	4	0.2	4	0.2	0	0.0	0.45
ABO mismatch	Matched	1114	53.0	888	53.3	226	52.0	
	Minor	389	18.5	305	18.3	84	19.3	
	Major	354	16.8	287	17.2	67	15.4	
	Both	186	8.9	148	8.9	38	8.7	
	Unknown	59	2.8	39	2.3	20	4.6	0.12
GVHD prophylaxis	CyA based	1033	49.1	816	49.0	217	49.9	
	Tac based	1069	50.9	851	51.0	218	50.1	0.73
Year of HCT	≤2008	1107	52.7	875	52.5	232	53.3	
	≥2009	995	47.3	792	47.5	203	46.7	0.75
Follow-up period	Median	1134		1130		1171.5		0.11
	(Range)	(40–4947)		(40–4922)		(41–4947)		

*CY* cyclophosphamide, *TBI* total-body irradiation, *HDCA* high-dose cytarabine, *PS* performance status, *HCT-CI* hematopoietic cell transplant co-morbidity index, *CMV* cytomegalovirus, *AML* acute myelogenous leukemia, *MDS* myelodysplastic syndrome, *Rel* related donor, *UR* unrelated donor, *BM* bone marrow graft, *PB* peripheral blood stem cell graft, *NCC* nucleated cell count, *HLA* human leukocyte antigen, *M to F* male to female, *F to M* female to male, *GVHD* graft-versus-host disease, *CyA* cyclosporine, *Tac* tacrolimus

^*^Indicates statistically significant by the *χ* ^2^ test or Student’s *t* test

**Fig. 1:**
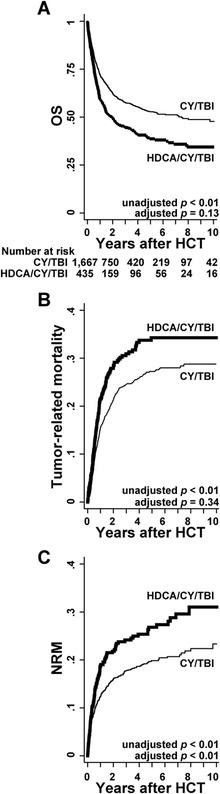
Prognosis after HCT in each group of the conditioning regimen. **a** OS was calculated with the Kaplan-Meier method in each group of HDCA/CY/TBI and CY/TBI. HR for overall mortality of HDCA/CY/TBI compared to CY/TBI was calculated by Cox proportional hazards model after being adjusted for confounding factors such as patient sex, age, PS, CMV sero-status, diagnosis, disease risk, days from diagnosis to HCT, HLA mismatch, sex mismatch, and year of HCT. **b** Tumor-related mortality, defined as death without remission or after relapse, was calculated using Gray’s method considering therapy-related death as a competing risk. HR was calculated by using Fine-Gray proportional hazards model adjusted by the confounding factors mentioned above. **c** NRM was calculated using Gray’s method considering relapse as a competing risk. HR was also calculated using the same model

OS of the HDCA/CY/TBI group was inferior to that of the CY/TBI group (Fig. 1a; 59.3 vs 72.0 % at 1 year; 45.3 vs 58.8 % at 3 years after HCT). This difference was significant on univariate analysis (Table 2), but not on multivariate analysis after adjustment for confounding factors (hazard ratio (HR), 1.14; *p* = 0.13; Table 3). In subgroup analyses according to pre-transplant characteristics, OS in the HDCA/CY/TBI group was significantly inferior in standard-risk disease (HR, 1.52; *p* < 0.01). No significant differences were found in other subgroups (Additional file 1: Figure S1).

**Table 2:** Univariate analysis of prognosis

Variables		Overall mortality		
		HR	(95 % CI)	*p*
Conditioning	CY/TBI	(Reference)		
	HDCA/CY/TBI	1.50	(1.29–1.74)	<0.01^*^
Other variables				
Sex	Female	(Reference)		
	Male	1.00	(0.88–1.14)	0.96
Age	≤49	(Reference)		
	≥50	1.93	(1.65–2.25)	<0.01^*^
PS	0–1	(Reference)		
	≥2	2.20	(1.74–2.80)	<0.01^*^
HCT-CI	≤2	(Reference)		
	≥3	1.90	(1.46–2.47)	<0.01^*^
CMV sero-status	Negative	(Reference)		
	Positive	1.24	(1.04–1.48)	0.02^*^
Diagnosis	AML	(Reference)		
	MDS	0.61	(0.49–0.75)	<0.01^*^
Disease risk	Standard	(Reference)		
	High	2.38	(2.10–2.71)	<0.01^*^
Days from diagnosis to HCT	≤240	(Reference)		
	≥241	0.89	(0.78–1.01)	0.08
Donor	Rel-BM	(Reference)		
	Rel-PB	1.16	(0.96–1.40)	0.13
	UR-BM	1.11	(0.95–1.31)	0.20
HLA mismatch	Matched	(Reference)		
	Mismatched	1.25	(1.01–1.43)	<0.01^*^
Sex mismatch	Matched	(Reference)		
	M to F	1.04	(0.89–1.22)	0.60
	F to M	1.16	(0.99–1.36)	0.07
ABO mismatch	Matched	(Reference)		
	Minor	0.90	(0.75–1.07)	0.23
	Major	1.11	(0.93–1.32)	0.23
	Both	0.83	(0.64–1.07)	0.15
GVHD prophylaxis	CyA based	(Reference)		
	Tac based	1.01	(0.89–1.15)	0.87
Year of HCT	≤2008	(Reference)		
	≥2009	0.86	(0.75–0.99)	0.04^*^

Other abbreviations are explained in Table 1

*HR* hazard ratio, *CI* confidence interval

^*^Indicates statistically significant

**Table 3:** Multivariate analysis of prognosis in patients with HDCA/CY/TBI compared with CY/TBI

Variables		Overall mortality				Tumor-related mortality				NRM			
		HR		(95 % CI)	*p*	HR		(95 % CI)	*p*	HR		(95 % CI)	*p*
Conditioning	CY/TBI	(Reference)				(Reference)				(Reference)			
	HDCA/CY/TBI	1.14	(0.96–1.34)		0.13	0.90		(0.72–1.12)	0.34	1.48		(1.15–1.91)	<0.01^*^
Other variables													
Age	≤49	(Reference)				(Reference)				(Reference)			
	≥50	1.86	(1.57–2.20)		<0.01^*^	1.31		(1.02–1.68)	0.03^*^	2.04		(1.60–2.61)	<0.01^*^
PS	0–1	(Reference)				(Reference)				(Reference)			
	≥2	1.42	(1.09–1.85)		<0.01^*^	1.73		(1.25–2.39)	<0.01^*^	0.71		(0.41–1.23)	0.22
CMV sero-status	Negative	(Reference)				(Reference)				(Reference)			
	Positive	1.12	(0.93–1.34)		0.23	1.29		(1.00–1.65)	0.05^*^	0.91		(0.70–1.18)	0.47
Diagnosis	AML	(Reference)				(Reference)				(Reference)			
	MDS	0.40	(0.32–0.51)		<0.01^*^	0.18		(0.12–0.27)	<0.01^*^	1.18		(0.87–1.59)	0.28
Disease risk	Standard	(Reference)				(Reference)				(Reference)			
	High	2.53	(2.18–2.93)		<0.01^*^	3.98		(3.26–4.85)	<0.01^*^	0.97		(0.77–1.23)	0.82
Days from diagnosis to HCT	≤240	(Reference)				(Reference)				(Reference)			
	≥241	0.88	(0.76–1.01)		0.07	0.80		(0.66–0.97)	0.02^*^	0.98		(0.79–1.22)	0.89
HLA mismatch	Matched	(Reference)				(Reference)				(Reference)			
	Mismatched	1.25	(1.08–1.44)		<0.01^*^	0.90		(0.73–1.10)	0.30	1.60		(1.29–1.99)	<0.01^*^
Sex mismatch	Matched	(Reference)				(Reference)				(Reference)			
	M to F	0.97	(0.81–1.15)		0.70	0.91	(0.72–1.15)		0.44	1.01	(0.77–1.31)		0.97
	F to M	1.12	(0.94–1.33)		0.20	0.94	(0.74–1.20)		0.63	1.28	(0.99–1.65)		0.07
Year of HCT	≤2008	(Reference)				(Reference)				(Reference)			
	≥2009	0.89	(0.77–1.02)		0.10	0.96	(0.80–1.16)		0.70	0.76	(0.61–0.94)		0.01^*^

Other abbreviations are explained in Tables 1 and 2

*NRM* non-relapse mortality

^*^Indicates statistically significant

Relapse, tumor-related mortality, and NRM were calculated; relapse was not reduced by HDCA addition (HR, 0.90; 95 % confidence interval (CI), 0.63–1.30; *p* = 0.58), resulting in unmitigated tumor-related mortality in the HDCA/CY/TBI group (Fig. 1b and Table 3) regardless of disease risk (high risk: HR, 0.91; *p* = 0.47; standard risk: HR, 0.84; *p* = 0.46). On the other hand, HDCA/CY/TBI significantly increased NRM in the whole cohort (HR, 1.48; *p* < 0.01; Fig. 1c and Table 3) especially in the acute phase after HCT. The major causes of NRM included organ failure, infection, and GVHD, without significant differences between the two groups (Table 4).

**Table 4:** Causes of NRM

Cause of NRM	Total		CY/TBI		HDCA/CY/TBI		
	*N*	%	*N*	%	*N*	%	*p*
Infection	110	27.3	78	26.4	32	29.6	0.52
Bacteria	61		43		18		
Virus	19		14		5		
Fungi	13		9		4		
Rejection/engraftment failure	3	0.7	3	1.0	0	0.0	0.29
TMA	10	2.5	8	2.7	2	1.9	0.62
VOD	15	3.7	12	4.1	3	2.8	0.54
GVHD	44	10.9	32	10.8	12	11.1	0.94
Acute	20		14		6		
Chronic	24		18		6		
Hemorrhage	24	6.0	18	6.1	6	5.6	0.84
Organ failure	127	31.5	95	32.2	32	29.6	0.62
Liver	9		7		2		
Heart	13		8		5		
Kidney	6		4		2		
CNS	8		6		2		
Lung	82		62		20		
Interstitial pneumonia	40		29		11		
ARDS	14		13		1		
Secondary malignancy	1	0.2	1	0.3	0	0.0	0.54
Others	69	17.1	48	16.3	21	19.4	
Total	403	100.0	295	100.0	108	100.0	

Other abbreviations are explained in Tables 1–3

*TMA* thrombotic microangiopathy, *VOD* veno-occlusive disease, *CNS* central nervous system, *ARDS* acute respiratory distress syndrome

**Fig. 2:**
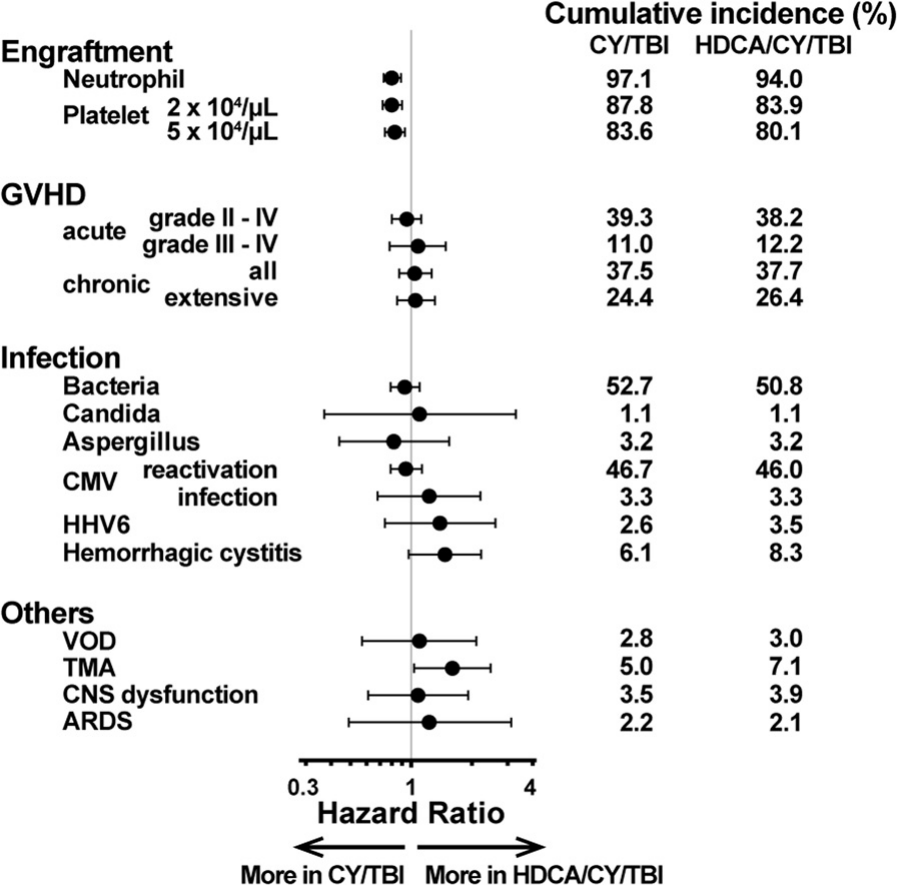
Clinical courses after HCT in each group of CY/TBI and HDCA/CY/TBI. The cumulative incidence of major clinical events after HCT, such as engraftment, GVHD, infection, and other acute phase complications are summarized. In each event, adjusted HRs in the HDCA/CY/TBI group were analyzed in comparison with the CY/TBI group. *Dots* indicate HRs, and *bars* indicate 95 % CI ranges

We compared the clinical courses that led to higher NRM in HDCA/CY/TBI, with a focus of engraftment, GVHD, infection, and other acute phase complications (Fig. 2). The HDCA/CY/TBI group showed significantly lower proportions of neutrophil and platelet engraftment following HCT (HR, 0.80; *p* < 0.01, and HR, 0.83; *p* < 0.01, respectively). Complete chimerism was achieved in 78.2 % of the CY/TBI group vs 72.6 % of the HDCA/CY/TBI group (*p* = 0.04). We observed no significant differences in the incidence of acute or chronic GVHD (grades II–IV acute GVHD, 39.3 vs 38.2 %; chronic GVHD 37.5 vs 37.7 %, respectively) (Fig. 2). Hemorrhagic cystitis, mostly due to viral reactivation or infection [11], and thrombotic microangiopathy (TMA) were more frequently observed in the HDCA/CY/TBI group (HR, 1.47; *p* = 0.06, and HR, 1.60; *p* = 0.04, respectively). These two complications were related to a significantly higher proportion of NRM (data not shown). Other potential complications of HDCA, such as central nervous system (CNS) dysfunction and acute respiratory dysfunction syndrome (ARDS) [12], were not increased in the HDCA/CY/TBI group.

## Discussion

In this study on myeloablative BMT/PBSCT for AML/MDS, we did not observe the improvement of OS in the HDCA/CY/TBI group due to (1) a higher proportion of NRM and (2) the lack of apparent additional anti-leukemic effect of HDCA. These results differ from those of CBT, in which a stronger anti-leukemic effect without increased NRM led to superior OS in HDCA/CY/TBI [5].

Among acute phase complications that can lead to NRM, hemorrhagic cystitis and TMA were increased after HDCA/CY/TBI. These complications, if not resolved early, can induce renal failure, prohibit immune reconstitution, and deteriorate patient’s nutrition and performance status, which may ultimately lead to significantly higher NRM [11, 13]. The strong cytotoxicity of HDCA combined with significantly poorer neutrophil engraftment might cause cystitis-related virus reactivation or vascular endothelial cell injury which can induce hemorrhagic cystitis or TMA. These features were not observed in CBT [5]; the relatively higher incidence of acute GVHD in BMT/PBSCT can explain this difference because both hemorrhagic cystitis and TMA are closely related to preceding acute GVHD [11, 13].

On the other hand, no additional anti-leukemic effect of HDCA was apparent in this study. HDCA can reduce the remaining leukemia cells that may cause relapse after HCT [12]. This anti-leukemic effect of HDCA directly reduced the incidence of relapse in CBT [5] because graft-versus-leukemia (GVL) effects after CBT was relatively weak [14]; relapse after CBT mainly depends on the efficacy of conditioning regimens [5]. In BMT/PBSCT, however, GVL effects are much stronger than CBT especially in the case of HLA-mismatched transplantation [14]. Suppression of total relapse after HCT is mainly attributed to continuous GVL effects [14] compared to the conditioning regimens which will be inactivated rapidly after HCT [12]; strength of conditioning regimens (for example, HDCA addition in this study) may not directly influence on relapse reduction. These differences in GVL effects can partly explain the discrepancy in the efficacy of HDCA on post-transplant relapse or disease-related death.

A larger proportion of patients with high-risk disease and worse performance status in the HDCA/CY/TBI group may confound the outcomes, but multivariate and subgroup analyses indicated unimproved prognosis in HDCA/CY/TBI even after eliminating those confounding factors. Moreover, subgroup analyses regarding the percentage of myeloblast just before conditioning regimens were carried out; no significant differences of OS between CY/TBI and HDCA/CY/TBI were found in any subgroups (data not shown). The bias in regard to the HCT centers, however, still remains to be overcome in this study. The choice of conditioning regimen depends on the attending physicians in each institution, indicating that the clinical experiences of each transplant center can be a confounding factor. Unfortunately, we were not able to adjust this factor because the database did not contain such information.

The combination of granulocyte colony stimulating factor (G-CSF) with HDCA is another important topic; it is reported that G-CSF-combined HDCA/CY/TBI provided low NRM and high OS in a previous study [15]. In our cohort, patients with G-CSF-combined HDCA/CY/TBI regimen (*N* = 25) revealed almost the same prognosis (HR, 1.02; 95 %CI, 0.59–1.76; *p* = 0.95) as HDCA/CY/TBI without G-CSF.

In summary, this study showed the inefficacy of adding HDCA to CY/TBI in BMT/PBSCT for AML/MDS, suggesting that the merits of HDCA in CBT cannot be extrapolated to BMT/PBSCT. Incidence of GVHD or strength of GVL effects may be related to these differences between donor sources. This single-country retrospective analysis should be validated in future prospective studies in order to determine proper conditioning regimens in BMT/PBSCT for AML/MDS.

## Additional file

Additional file 1: Figure S1. Subgroup analyses of OS with respect to patient characteristics. OS was compared in each subgroup with respect to patient characteristics. The adjusted HRs of overall mortality in the HDCA/CY/TBI group were shown compared to the CY/TBI group. Black dots indicate HRs, and 95 %CI ranges are shown by black bars.

## Abbreviations

AML: acute myelogenous leukemia
BMT: bone marrow transplantation
CBT: cord blood transplantation
CI: confidence interval
CY: cyclophosphamide
GVHD: graft-versus-host disease
GVL: graft versus leukemia
HCT: hematopoietic cell transplantation
HDCA: high-dose cytarabine
HLA: human leukocyte antigen
HR: hazard ratio
MDS: myelodysplastic syndrome
NRM: non-relapse mortality
OS: overall survival
PBSCT: peripheral blood stem cell transplantation
Rel: related donor
TBI: total-body irradiation
TMA: thrombotic microangiopathy
UR: unrelated donor
