# Supplementary material for: Clinical significance of high-dose cytarabine added to cyclophosphamide/total-body irradiation in bone marrow or peripheral blood stem cell transplantation for myeloid malignancy
Source: J Hematol Oncol. 2015 Sep 4;8:102. doi: 10.1186/s13045-015-0201-x (PMC4559384; doi:10.1186/s13045-015-0201-x)
Supplement: Additional file 1: Figure S1. — Subgroup analyses of OS with respect to patient characteristics. OS was compared in each subgroup with respect to patient characteristics. The adjusted HRs of overall mortality in the HDCA/CY/TBI group were shown compared to the CY/TBI group. Black dots indicate HRs, and 95 %CI ranges are shown by black bars. [file 13045_2015_201_MOESM1_ESM.pdf]

Supplemental Figure 1

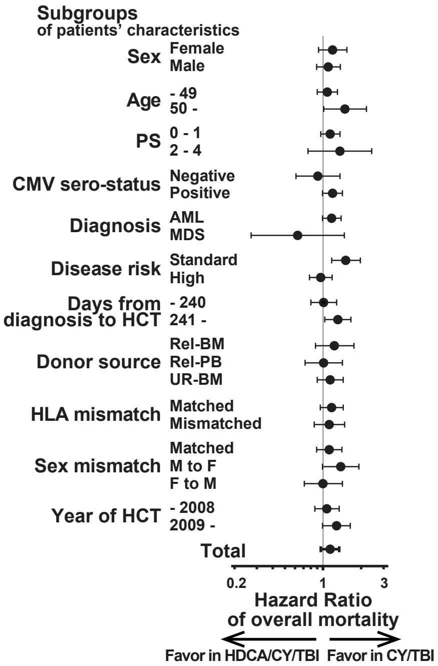

Subgroup analyses of OS with respect to patient characteristics
OS was compared in each subgroup with respect to patient characteristics. The adjusted HRs of overall mortality in the HDCA/CY/TBI group were shown compared to the CY/TBI group. Black dots indicate HRs, and 95%CI ranges are shown by black bars.
